# The Impact of Salts on Single Chain Amphiphile Membranes and Implications for the Location of the Origin of Life

**DOI:** 10.3390/life7040044

**Published:** 2017-11-14

**Authors:** Sarah Maurer

**Affiliations:** Department of Chemistry and Biochemistry, Central Connecticut State University, 1615 Stanley St., New Britain, CT 06050, USA; smaurer@ccsu.edu; Tel.: +1-860-832-2575

**Keywords:** prebiotic, vesicles, liposomes, protocell, abiogenesis, ionic solutes, amphiphiles

## Abstract

One of the key steps in the origins of life was the formation of a membrane to separate protocells from their environment. These membranes are proposed to have been formed out of single chain amphiphiles, which are less stable than the dialkyl lipids used to form modern membranes. This lack of stability, specifically for decanoate, is often used to refute ocean locations for the origins of life. This review addresses the formation of membranes in hydrothermal-vent like conditions, as well as other environmental constraints. Specifically, single chain amphiphiles can form membranes at high sea salt concentrations (150 g/L), high temperatures (65 °C), and a wide pH range (2 to 10). It additionally discusses the major challenges and advantages of membrane formation in both ocean and fresh water locations.

## 1. Early Earth Environments for the Origins of Life

Life is hypothesized to have originated on Earth between 4.2 bya and 3.8 bya [1,2]. Energy, water, and chemicals would have had to combine in perhaps precise amounts and sequences for this process to have occurred. As these environments are reviewed elsewhere in this issue [3], as well as in other sources, they will only briefly be reviewed here.

Hydrothermal vents are often proposed and studied as early locations for abiogenesis [4,5,6]. These deep ocean locations provide the right environments and reactants for many reactions that lead to the molecules used in modern biology [7,8,9]. This is due to the temperature, pressure, water, mineral surfaces, and simple chemicals available in these environments. However, vast oceans may dilute any products formed, and the high concentration of salt in these locations make them untenable for certain prebiotic evolutionary models.

Inland locations with access to fresh water, likely through precipitation, are also possible locations for the origin of life [10,11]. These locations could have chemical precursors available from meteoric sources, and perhaps geothermal systems, with lower concentrations of dissolved ionic solutes and dehydration as a chemical driving force for non-enzymatic polymerization reactions [12,13]. However, these regions are thought to have had lower concentrations of high energy chemicals than hydrothermal systems and would be exposed to harsh UV irradiation.

One of the main arguments used to disfavor hydrothermal vent environments is the conjecture that the primitive membranes that formed the first cell-like structures could not form due to high (divalent) salt concentrations [11,14]. We disagree: membranes composed of prebiotic amphiphiles benefit from increases in ionic strength, and prebiotic vesicles can be formed in a high ionic strength solutions, including seawater (containing divalent cations), making hydrothermal environments plausible locations for the first cell membranes to have formed. Here we present a review of these membranes and hope to steer the abiogenic environment discussion away from primitive membrane stability to more relevant topics.

## 2. Membranes and Life

Membranes serve many purposes in a cell and are considered a requirement for the formation of life. These amphiphilic bilayers primarily act as a hydrophobic barrier between the environment and the cytosol, preventing the diffusion of molecules into and out of the cell. They additionally can contain energy harvesting molecules like the proteins in the electron transport chain required for oxidative phosphorylation and maintain the chemical gradients necessary to use these molecules. Finally, membranes serve as a platform for evolution by defining an individual within a population for selection to act upon.

Primitive membrane models have been shown to perform many of these ‘living’ functions, as described above [15,16,17,18]. Membranes composed of just ten-carbon amphiphiles have been shown to encapsulate template directed synthesis of nucleic acids from externally added nucleotides [19]. Other biologically relevant reactions have also been demonstrated in such systems, including the polymerization of nucleic acids through dehydration, catalysis using ribozymes, and catalysis using proteins [12,20,21]. The involvement of these membranes in energy generation (i.e., photochemistry) is also well established [22,23,24,25,26]. Additionally, these membranes display growth and division cycles, allowing for the replication of primitive cells [27,28,29,30]. The research to date shows these simple membranes to be versatile components of model proto-cells.

Early membranes are proposed to have been made of chemically simpler molecules than modern cell membranes while still performing the above functions [31]. Modern membranes are composed primarily of dialkyl amphiphiles with a charged head group. However, monoalkyl amphiphiles are more likely to have formed from prebiotic chemical synthesis, including hydrothermal [7,8,32,33] and interstellar reactions [34,35].

Using the top down approach to help discern the origins of life, we can gain insight into primitive membranes by examining modern membranes. Using this approach, fatty acids are often cited as the most likely primitive amphiphile [36,37]. If modern bacterial membranes evolved from primitive membranes, the first amphiphile contained an alkyl hydrophobic group and a carboxylate head group, generating a fatty acid. These fatty acids could then have later condensed onto a glycerol backbone to form the more stable diacyl amphiphiles. However, fatty acids may also have been a later adaptation that was advantageous for other reasons (e.g., energy storage), and this approach is not the only means of unraveling life’s origins.

Using a bottom up approach, very different conclusions can be made. The prebiotic chemical library, generated from hydrothermal, atmospheric, meteoric, and perhaps other sources, was a mix of chemicals with larger abundances of simple small molecules and lower abundances of more complex, larger molecules [8,38]. These mixtures were unlikely to have any one amphiphile, and a wide range of functional groups can be found in these mixtures, including amines, alcohols, aldehydes, and carboxylates, among others. If the first cells were assembled from environmentally available amphiphiles, this would indicate that a mixture of amphiphiles would have formed the first cell membranes.

Generally, the bottom up and the top down approach meet in the middle, where a successful replicator is thought to adopt a single strategy. Therefore, while carboxylates were most likely part of the first membranes, it is unlikely they were the only amphiphile present.

## 3. Salt and Membranes

Using a top-down approach to determine the salinity of the first cells, neither sea water nor fresh water closely mimics contemporary cytosol (Table 1). Single celled organisms have cytosol that is highly dependent on growth media, making this task more difficult. It is also not known how contemporary cytosol differs from the cytosol of the last universal common ancestor (LUCA).

Fresh water was likely available from precipitation and dew formation but was by far less prevalent, as the continental mass was even smaller than it is today. All cells contain relatively concentrated ionic solutions compared to fresh water, freshwater seems an unlikely choice for the formation of life, especially considering the advantages hydrophobic self-assembly has in higher ionic strength solutions [39,40].

Seawater is much higher in total ionic strength than cytosol (>3 times), and the ratios more closely mimic blood (or extracellular environment), with high concentrations of sodium but low concentrations of potassium. Additionally, the concentration of ions in the early oceans was likely equal, if not higher than, in the modern oceans, although the ions present may have differed due to the lack of oxygen in the atmosphere and the temperature, among other factors [41]. The water present on early Earth quickly equilibrated with the atmosphere, and the mineral surfaces present to generated oceans with high ionic strength, likely 1.6 to two times saltier than today’s oceans. This makes seawater an unlikely choice for the first cytosol as well.

The first primitive membranes were reported in 1978 by Hargreaves and Deamer, demonstrating that short, saturated fatty acids and fatty acid/alcohol mixtures could self-assemble into membranes [36]. These amphiphiles were appealing, not only because they are simpler than phosphoglycerolipids, but also a chemical building block making fatty acids a clear precursor to modern lipids. Since many properties of these membranes have been explored, it is clear that they are capable of a range of ‘living’ functions [14,21,22,23,28,30].

However, carboxylates precipitate in the presence of divalent cations, specifically Mg^2+^ and Ca^2+^, which are present in seawater. To form membranes, fatty acids must be partially deprotonated (close to their p*K_a_*); therefore the membranes will precipitate in ocean-like conditions. Additionally, divalent cations are a requirement for many nucleic acid reactions, making these membranes less compatible with the RNA world hypothesis.

While many articles discuss the incompatibility of high ionic strength solutions with membranes, few discuss the need for salt in single chain amphiphile membrane formation [39,45]. The ionic strength of fresh water would likely be insufficient to form membranes from decanoic acid, as about 10 mM of salt is needed to find vesicles. Most experiments using fatty acid membranes do not report NaCl addition, as it is added inadvertently through pH vesiculation: by increasing the pH with NaOH then decreasing the pH to the p*K_a_* of the fatty acid with HCl [46]. This formation improvement is likely caused by both salting-in the hydrophobic tails and charge-shielding the carboxylate headgroups, allowing for the formation of fatty acid membranes [40,47].

**Figure 1:**
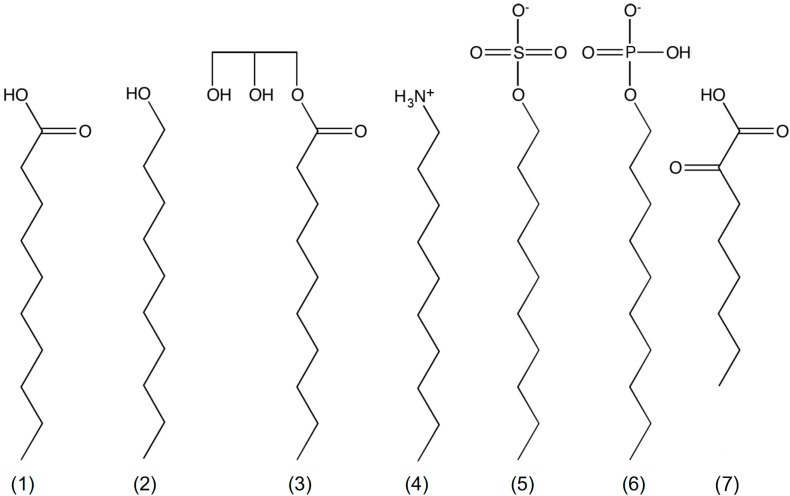
Chemical structures of mono decyl amphiphiles. (1) decanoic acid; (2) decanol; (3) glycerol monodecanoate; (4) decylamine; (5) decylsulfate; (6) decylphosphate; and (7) 2-ketooctanoic acid.

Many simple membrane models have now been proposed from a range of single chain amphiphiles (Figure 1) [48,49,50,51,52,53,54,55]. Membranes composed of non-fatty acid amphiphiles or mixtures of fatty acids with other amphiphiles were found to be more stable than fatty acid/carboxylate membranes; they showed less divalent salt sensitivity [14,55], higher critical vesicle concentrations [22,48], more pH-range resilience [14,49,55], and less mixing between populations [48]. These amphiphiles include the often-used long chain alcohols and glycerol monoacylates but also less commonly reported charged groups like alkyl phosphates, ketocarboxylic acids, and amines.

It is likely that a wide variety of membranes need some amount of salt to form, and the author therefore recommends adding a fixed amount of saline to buffers to aid in aggregation (30 to 100 mM). While decanoic acid/decanoate membranes tend to ‘salt-out’ of solution around 200 mM NaCl, mixtures of single chain amphiphile membranes are often stable at much higher concentrations of salt [39]. 

**Figure 2:**
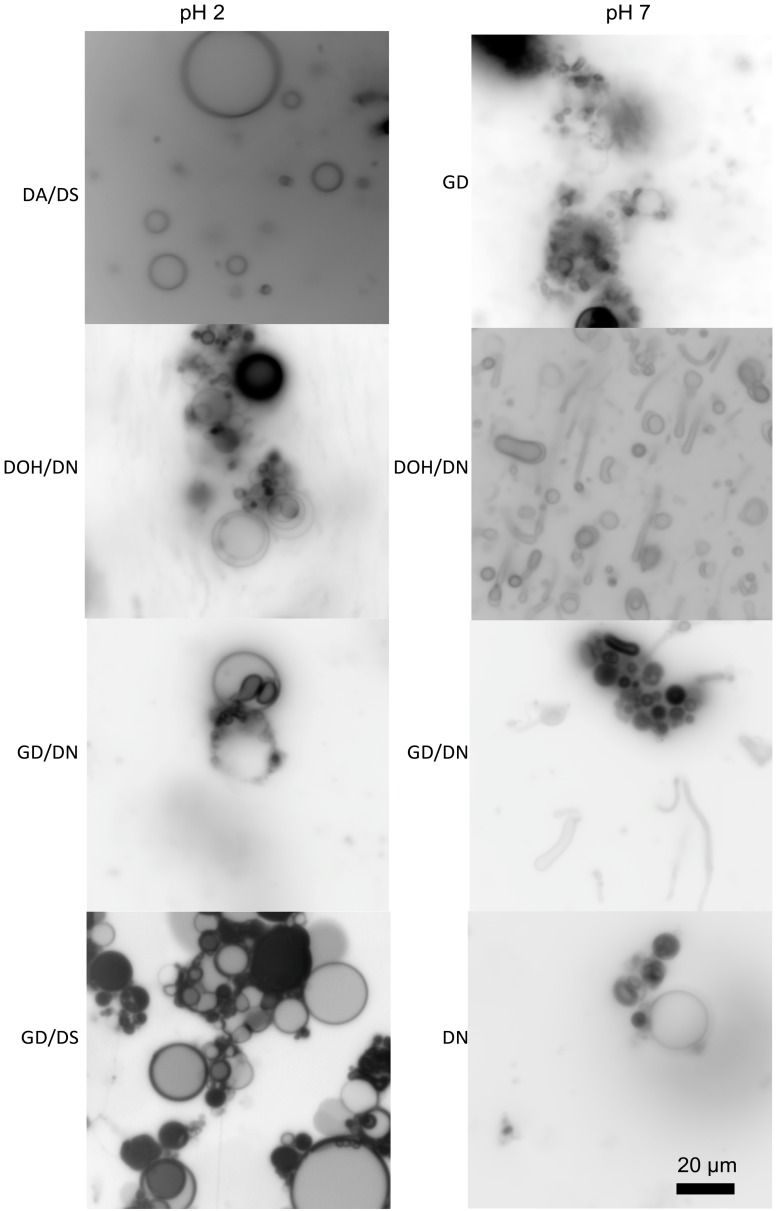
Membrane formation of various amphiphiles in the presence of 35 g/L sea salt. The abbreviations used are decanoic acid (DA), decylsulfate (DS), decanol (DOH), decylamine (DN), and glycerol monodecanoate (GMD). The total concentration of lipid in each sample is 25 mM, and the mixtures of amphiphiles are in a 1:1 ratio. Original micrographs are from the author. The scale bar is the same for all micrographs.

**Figure 3:**
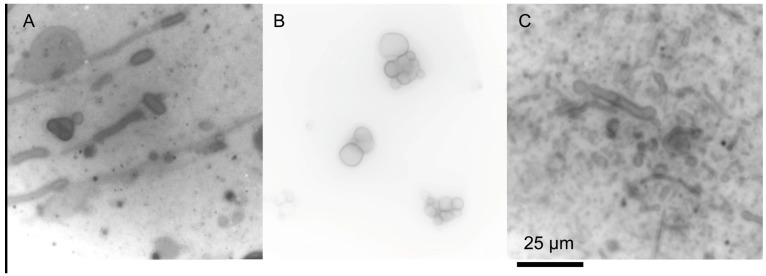
Decanoic acid/decylamine mixtures (equimolar, 25 mM total concentration) at higher concentrations of sea salt. (**A**) 60 g/L of sea salt at room temperature. (**B**) 150 g/L sea salt at room temperature. (**C**) 150 g/L sea salt at 65 °C. Original micrographs are from the author. The scale bar is the same for all micrographs.

Some mixtures of single-chain amphiphiles can form stable membranes in seawater solutions. Namani and Deamer originally showed the formation of decanoic acid/decylamine vesicles in the presence of seawater [51]. Additionally, ketocarboxylic acids and phosphate vesicles are stable in seawater solutions [49,55]. Recently, we have found that seawater can be used to form membranes out of a variety of amphiphile mixtures (Figure 2) [56]. The clear exception is carboxylates, which precipitate due to the Mg^2+^ content, but, if the pH is kept relatively low, even fatty acids can be included in the mixture. The concentration of the seawater can even be increased above Archean estimates, and membranes of decanoic acid/decylamine will still form (Figure 3A) [56]. Additionally, mixtures of amphiphiles would have been far more likely in the absence of a specific enzymatic synthesis, reducing the carboxylate precipitation problems commonly cited as a barrier to a saltwater/hydrothermal origin.

## 4. Other Environmental Factors

Simple membranes are impacted by other environmental conditions, although each factor’s impact is specific to the composition of the amphiphile in the membrane. For example, some compositions are only stable as membranes at low temperatures, while others only form membranes at high temperatures [48,57]. This is means that there are compositions of single chain amphiphiles that can form membranes under most conditions, but a composition of single chain amphiphiles that forms membranes under one extreme condition is unlikely to form membranes under different conditions. Hydrothermal environments have many ‘extreme’ conditions, but it is this author’s conjecture that perhaps it is not truly impossible to find an amphiphile composition that will form membranes.

The pH of contemporary oceanic vents can vary from 2 to 3 in black smoker chimneys [58] to a much higher pH of almost 10 in white smoker chimneys [59], compared to an ocean pH of about 8. Some single chain amphiphiles are known to form membranes under these conditions. Decylphosphate was capable of forming membranes at low pH [49,60], and mixtures of decylamine with a wide range of co-surfactants produce membranes that are pH stable at both low and high pH, even in the presence of sea salt ([51,56]). However, if we assume that fatty acids alone were the precursor to modern membranes, the pH of the solution in which membranes form would be dependent on the hydrocarbon tail length of the fatty acid and likely be between 6 and 9 [22]. River pH is generally between these values [61]; cytosolic pH in human cells is generally 7.2 [42]. 

All membranes, even biological membranes, have temperature dependent formation. When the temperature is reduced below the phase transition temperature, the membranes lose integrity, becoming leaky, and preventing the function of membrane bound protein components (although the phase transition temperature of palmitoyl oleoyl phosphatidylcholine (POPC) is −2.5 °C [62]). Single chain amphiphile membranes are often more instable, precipitating and sometimes crystallizing if the temperature is decreased. Upon heating, these membranes can return to solution. Additionally, as the temperature is increased, the solubility of the amphiphiles increases, increasing the critical vesicle concentration, which dissolves the membranes. It has also been shown that increasing temperatures increases membrane fusion between individual vesicles [35]. White smoker hydrothermal vents are generally between 40 °C to 70 °C, while black smokers are much hotter, measuring between 60 °C to 400 °C. The ocean floor in these regions is generally around 2 °C, giving a gradient of temperatures around a vent system. Single chain amphiphile membranes can be observed at temperatures above room temperature. For example, dodecanoic acid/glycerol dodecanoate mixtures have been used to encapsulate fluorescent molecules at 42 °C [57]. Additionally, mixtures of decanoic acid/decylamine have been observed to form membranes at 65 °C (Figure 3C), and it is likely that other membranes are stable at these higher temperatures as well. The temperature may have fluctuated more rapidly in surface environments, where weather and day/night cycles would have a much greater impact.

Perhaps the greatest obstacle to the formation of membranes under prebiotic conditions is the accumulation of amphiphiles in sufficient concentrations to form membranes. For amphiphile molecules to accumulate, they either must be continuously synthesized in sufficient numbers to compensate for dilution or aggregate in a specific location. Synthesis in tectonic fault zones, for example, could account for the continuous release of amphiphiles into a prebiotic ocean [63]. In contrast, the build-up of amphiphiles in continental locations could occur by washing materials into natural pools that concentrate amphiphiles through dehydration [10]. However, this would also concentrate any other materials that are with the amphiphiles, like salts or oily contaminants, creating new challenges. Issues with low concentrations of amphiphiles are also mitigated by more complex mixtures of surfactants, which have lower critical vesicle concentrations [48,51,64]. With regards to generating sufficient concentrations of amphiphiles, the plausibility of either ocean or land origins are suspect; both locations are problematic in their own way.

The final environmental factor often considered in amphiphile chemistry is the presence of a mineral surface. The diversity of surfaces considered, both submarine and terrestrial, results in some surfaces being detrimental to membrane formation [65], while others are beneficial [66]. In ocean environments, the surface could play a lesser role, but most hydrothermal locations are ideal because of their mineral chemistry, so it is unlikely that the first cells would be removed too far from the often-catalytic locales. Therefore, both ocean and land locations have similar mineral challenges for vesicle formation.

## 5. Conclusions

Membranes are an essential component of cells, even protocells; therefore the environment for the origins of life must have permitted membrane formation to occur. Because decanoate, which precipitates in the presence of divalent cations, is often thought of as the precursor to modern membranes, ocean environments are sometimes referred to as unfavorable for membrane formation. The missing logic in this argument is that decanoate is only one among many possible early membrane-forming molecules, and mixtures of amphiphiles, even containing decanoate, are stabilized against divalent cation precipitation. In fact, membrane formation is often enhanced in the presence of salts due to changes in hydrophobicity and interactions between salts and charged amphiphile headgroups. 

By varying the composition of single chain amphiphile mixtures, it is possible to find stable membranes in most environmental conditions suggested for the origins of life. Therefore, the ability of a specific amphiphile (e.g., decanoate) to form membranes in an environment should not be used to determine the suitability of that environment for abiogenesis. Both ocean and land environments pose challenges to membrane formation but none that are truly insurmountable. Indeed, membrane formation through the self-assembly of single chain amphiphiles may be the easiest step in the formation of a functional biological cell.

## Figures and Tables

**Table 1:** Concentration of ions in relevant solutions.

Ion	Cytosol (mM) [42] *^a^*	Cytosol (mM) [43] *^b^*	Blood (mM) [43] *^b^*	River Water (mM) [44]	Seawater (mM) [14]
Potassium	200–250	139	4	0.051	10
Sodium	5	12	145	0.261	400
Chloride	6	4	116	0.226	460
Bicarbonate		12	29	0.951	2.1
Magnesium(free)	1–2	0.8	1.5	0.165	50
Calcium (free)	0.1	<0.0002	1.8	0.374	10
Sulfate				0.115	20
Other				0.233	0.5

*^a^* Values based on *E. coli*, which are dependent on growth media; *^b^* values based on humans.
